# Bone measurements interact with phenotypic measures in canine Duchenne muscular dystrophy

**DOI:** 10.3389/fvets.2024.1339833

**Published:** 2025-01-06

**Authors:** Sarah M. Schneider, Macie L. Mackey, Savannah Wilkinson, Lee-Jae Guo, Peter P. Nghiem

**Affiliations:** ^1^College of Veterinary Medicine and Biomedical Sciences, Texas A&M University, College Station, TX, United States; ^2^Department of Veterinary Integrative Biosciences, Texas A&M University, College Station, TX, United States

**Keywords:** Duchenne muscular dystrophy, golden retriever muscular dystrophy, GRMD, computed tomography, bone, muscle, dog, strength

## Abstract

Duchenne muscular dystrophy (DMD) is an X-linked muscle disease with weakness, loss of ambulation, and premature death. DMD patients have reduced bone health, including decreased femur length (FL), density, and fractures. The *mdx* mouse model has paradoxically greater FL, density, and strength, positively correlating with muscle mass. Bone morphology has not been extensively studied in the genetic homolog, golden retriever muscular dystrophy (GRMD). The aim of this study was to compare bone and muscle characteristics in GRMD dogs to understand their relationship to muscle function and density. We hypothesized that GRMD bone measurements would be altered similarly to DMD and would correlate with muscle strength and density. Eighteen variably aged and mixed gender dogs (6 each dystrophic, carrier, normal) were studied by computed tomography (CT) and bone measures were compared with various muscle functional measurements. FL, density, and volume and muscle density of several pelvic limb muscles were assessed. Dystrophic dogs showed some boney and muscle density changes on CT analysis compared to carriers and normal dogs. In GRMD, bone measurements were highly correlated with several other functional outcome measures, including eccentric contraction decrement, hip joint angle, and muscle volume/density. This novel experiment demonstrates an impact of dystrophy on bone outcome measures and provides observations on their correlation with functional outcomes.

## 1 Introduction

Duchenne muscular dystrophy (DMD) is an X-linked recessive, degenerative muscle disease affecting ~1 in 5,000 boys (1). The condition is caused by a genetic mutation in the *DMD* gene, followed by loss of the structural protein dystrophin. Lack of dystrophin leads to repeated cycles of muscle fiber necrosis and regeneration, with eventual muscle fiber replacement by fibrous connective tissue and fat (2). Affected boys experience loss of ambulation between 7 and 13 years (3) and cardiopulmonary failure and death in the third and fourth decade of life.

Dogs with genetically homologous golden retriever muscular dystrophy (GRMD) develop a similar progressive disease, the clinical course of which, in the first 6 months of life parallels the development of DMD over the first two decades (3). In particular, GRMD is characterized by progressive muscle degeneration and necrosis, connective tissue proliferation, and immune cell infiltration similar to DMD (3, 4). Flexor muscles tend to be affected early in life when the animal is crawling while extensors are affected after the animals begin walking. Interestingly, GRMD dogs tend to stabilize after the first 6 months, while DMD patients continue to progress in disease.

The repeated muscle damage, wasting and eventual failure to regenerate lead to progressive muscle weakness and muscle functional testing tracks and correlates with disease progression in DMD and GRMD (3, 4). In GRMD, tibiotarsal joint angles (joint contracture biomarker) and tetanic extensor torque (muscle strength biomarker) are greater (less affected) in mildly affected dogs than more severely affected dogs, while cranial sartorius (CS) circumference (muscle size biomarker) is the opposite, being larger in more severely affected dogs (3, 5–7). Phenotypic severity among different muscle groups, with some showing paradoxical hypertrophy (CS muscle) while others waste away, contributes to postural deficits and disability in both DMD and GRMD (8, 9). With regards to carriers and disease progression, it should be noted that carriers are female and typically do not show skeletal muscle signs, but human and canine carriers can develop cardiomyopathy in adulthood (10, 11).

Reduced bone density (osteoporosis) and fractures are a problem for boys with DMD. Some bone loss occurs secondary to loss of ambulation, particularly in the lumbar spine, but bone density in the femurs is markedly reduced in DMD prior to this (12). Other factors such as reduced muscle strength, lower vitamin D levels, and glucocorticoid treatment may contribute to decreasing bone density and a higher incidence of distal extremity fractures (12). Long bones of the appendicular skeleton were the most common fracture sites, with the femur, tibia, humerus, and clavicle having rates of 40, 26, 14 and 9%, respectively; lumbar compression fractures were relatively rare (12). Similarly, bone density and resistance to mechanical stress are initially reduced in femurs of the *mdx* mouse model of muscular dystrophy (13). However, in older *mdx* mice, density but not cortical thickness was recovered, and by 4 months *mdx* mice had higher bone mineral density and resistance to fractures along with increased hindlimb muscle mass (but not increased strength) (14, 15). The effects of dystrophinopathy on bone have not been extensively described or evaluated in dystrophic dogs. A single longitudinal study of bone mineral density using digital radiography with an aluminum stepscale to look at bone density in growing GRMD, carrier and normal dogs up to 9 months old was performed (16). The authors found significant differences between groups, with lower density in GRMD dogs (16).

We hypothesized that (1) GRMD dogs would have decreased bone density and shorter, thinner bones compared to normal and carrier dogs as measured by computed tomography (CT). We also hypothesized that (2) bone density and morphological measurements would correlate with muscle volume, density, and other functional outcome measures. Previous studies have demonstrated differences in muscle volume and density using histopathology and magnetic resonance imaging (MRI) between normal and GRMD dogs (17, 18), but have not included carriers. Finally, we hypothesized that (3) carrier dogs would be similar to normal dogs in bone and skeletal muscle volume and density as measured by CT.

## 2 Methods

### 2.1 Animals

All dogs {18 total; 6 each GRMD (2 female [F], 4 male [M]), carrier [6F], and normal [2F, 4 M]} were part of a GRMD dog colony cared for according to guidelines established by the National Research Council and in accordance with the Institute for Animal Care and Use Committee at Texas A&M University (2021–0105; Standard Operating Procedures-Canine X-Linked Muscular Dystrophy). Average ages were variable between the normal, carrier, and GRMD dogs were 17 [8–44], 41.7 [8–76], and 45 [8–77] months, respectively. Body weights (BW) were measured at CT imaging.

### 2.2 Computed tomography

Computed tomography data were derived from scans produced from a previously published study of glucose metabolism as a preclinical marker in GRMD in which the pelvic limbs were assessed by positron emission tomography (PET)/CT (19). As described in the original research (19), CT was performed on a 128-slice Siemens Biograph PET/CT scanner. For the current study, scout CT images were analyzed using the Inveon Research Workspace (Siemens Medical Solutions United States, Inc., Malvern, Pennsylvania). Full body scans were reoriented so that the transverse view was perpendicular to the femoral long axis and the sagittal and dorsal views were parallel to the sagittal and frontal femoral long axis. Computed tomography measurements of the femurs were made on both pelvic limbs and performed by one user (SW). Femur length (FL) was measured in a coronal view and cortical annular thickness (CorT) and cortical density (CorDen) were measured in the transverse plane.

#### 2.2.1 Femur length measurements

FL was measured along the long axis from the proximal tip of the greater trochanter to the level of the intercondylar notch, as described in a previous study (20). To perform the measurements, reoriented images needed to have an easily identified view of the entire greater trochanter, the majority of the femoral head, the widest part of the metaphyseal and diaphyseal medulla, and a clearly delineated intercondylar notch (or fossa). FL was measured in triplicate at the mid-dorsal slice and in slices 1 mm to either side and these three values were averaged.

#### 2.2.2 Cortical measurements

Next, CorT was measured in the transverse view at the mid-diaphyseal femur. Maximum (max) and minimum (min) CorT values were taken at the greatest and least diameters across the transverse section at the femoral midpoint. These values were derived by subtracting the medulla diameter from the overall diameter at the widest and narrowest points. The CorT measurements were performed in triplicates at the central slice, one slice above and one slice below, and the triplicate average was used for further analyses.

For CorDen measurements, due to our study limitations, we used CT and the Inveon Software program instead of the gold standard dual-energy X-ray absorptiometry (DXA) (21). CorDen was measured in the Inveon software by manual segmentation of the femur cortex in the same transverse section used for CorT measurement. CorDen was expressed as the average Hounsfeld units (HU, a measure of CT attenuation) within the region of interest (ROI).

#### 2.2.3 Muscle measurements

Using the CT scout scan, muscles were manually segmented at every 5th slice by a single viewer (SMS), and the whole muscle ROI was interpolated from these slices by the Inveon internal software (17, 19). Functional studies indicate that GRMD disease severity is similar for both pelvic limbs, so only one limb from each dog was examined (4). The mean HU and volume for each of the CS, vastus lateralis (VL), and rectus femoris (RF) muscles were automatically calculated by the program for each ROI.

### 2.3 Muscle strength measures

Tibiotarsal joint (TTJ) tetanic extension and flexion torque/force measurements, joint angles, eccentric contraction decrement (ECD), and surgically measured CS circumference were completed as previously described (4, 6, 22, 23) during a separate anesthetic event within 1–4 months after initial CT. Briefly, twitch and tetanic isometric muscle torque was assessed by percutaneously stimulating the fibular and tibial nerves to, respectively, flex and extend the TTJ muscles secured to a foot pedal that was attached to a transducer (Aurora Scientific). Force was calculated by normalizing to 0.75 X foot length in cm/body weight in kilograms. Eccentric contraction was performed by tetanic stimulation of the fibular nerve, flexing the muscles for 800 ms; on the final 200 ms, the foot pedal was simultaneously extended. Three reps of 10 eccentric contractions were performed, with 4 min rest in between each rep, for a total 30 replicates. Joint angles were measured with a goniometer while the animal was under general anesthesia in a resting, max flexed, and max extended position. During muscle biopsy of the CS muscle, surgical suture (two replicates) were used to encircle the CS muscle to measure circumference.

### 2.4 Statistical analysis

The statistical packages within GraphPad Prism (GraphPad Software) were used for data analysis. The significance level was set at a 2-tailed *p* value of <0.05. All absolute results were expressed as the mean (min/max). All data were checked for normality and, if necessary, converted logarithmically prior to statistical testing. We did not remove any possible outliers. We also did not do any imputation for missing data. We performed ANCOVA linear multi-regression analysis with the covariates’ genotype, age, sex, weight, and the interaction between genotype and sex. Values were collected from interpolated correction from ANCOVA (mean, upper/lower limit) and graphed. Normalized correlations (normalized for FL and BW) were reported as Pearson R coefficients, and *p* values were adjusted for multiple testing.

## 3 Results

### 3.1 Age and body weight

Average ages were variable between the normal, carrier, and GRMD dogs at 17 [8–44], 41.7 [8–76], and 45 [8–77] months, respectively. Body weights (BW) were measured at CT imaging. In the variably aged and mixed gender groups, weight did not differ significantly between groups (Supplementary Figure 1).

### 3.2 Femoral length

**Figure 1:**
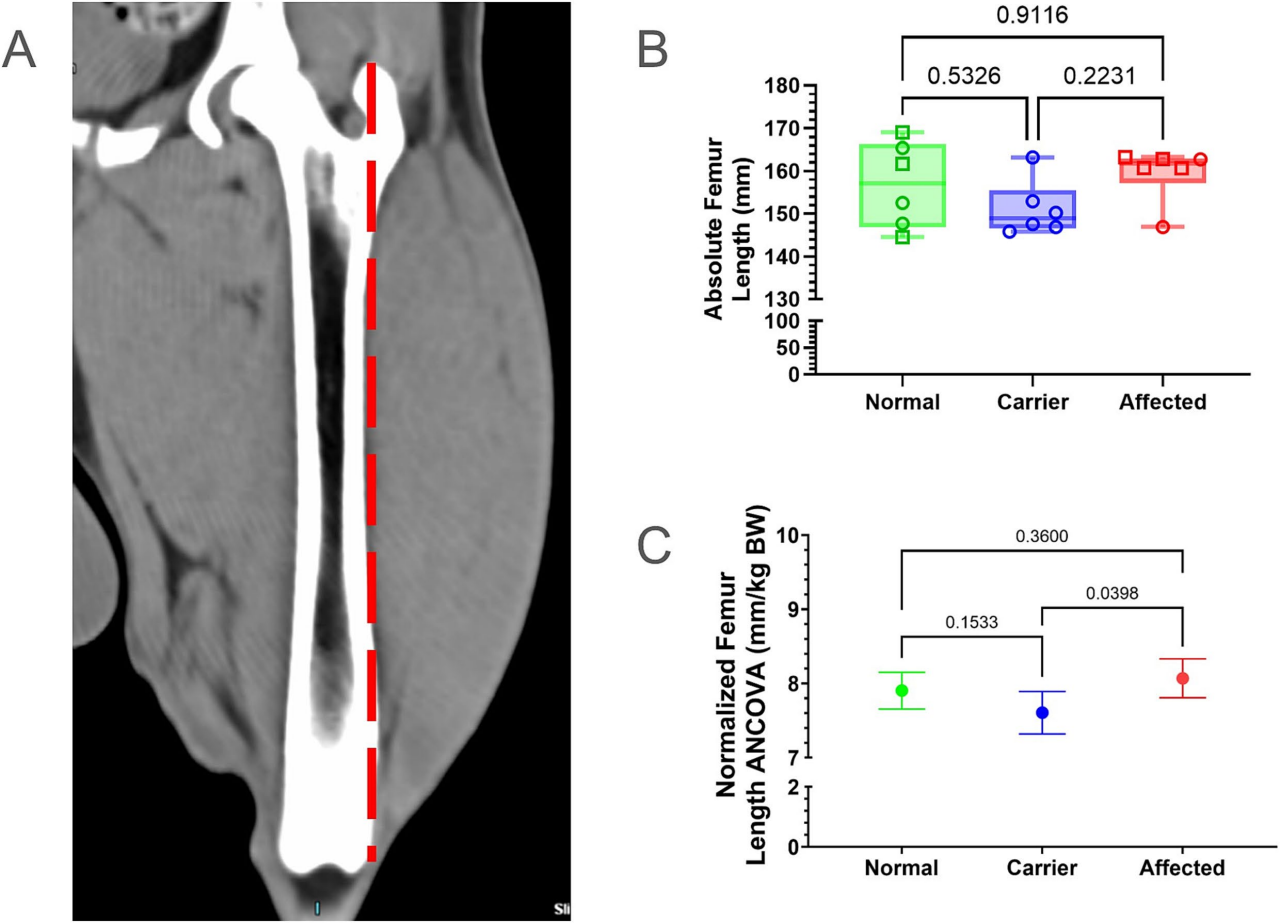
Femur length (FL) comparisons. **(A)** FL was measured from the proximal tip of the greater trochanter to the level of the intercondylar notch (dotted red line). **(B)** Raw values were in millimeters (mm); min, max, and mean. Results were analyzed via One Way ANOVA and plotted with individual dog values. KG, kilogram, BW, body weight, Green = Normal, Blue = Carrier, Red = GRMD affected dogs, male (square), female (circle). **(C)** Multiple linear regression (ANCOVA) plus a normality test (Gaussian) were performed, adjusting for covariates’ genotype, weight, age, and sex. The interpolated mean was graphed and included min and max.

Absolute FL was measured from the proximal tip of the greater trochanter to the level of the intercondylar notch (Figure 1A); femoral measurements were normalized to BW. For the most part, absolute and normalized femoral measurements tracked with one another (Supplementary Table 1; Figures 1B,C). Normal dogs did not differ from GRMD nor carrier dogs in absolute or normalized FL. However, GRMD dogs had greater normalized FL than carriers (*p* < 0.05; Figure 1C).

### 3.3 Mid-diaphyseal cortical annular thickness of the femur (CorT)

**Figure 2:**
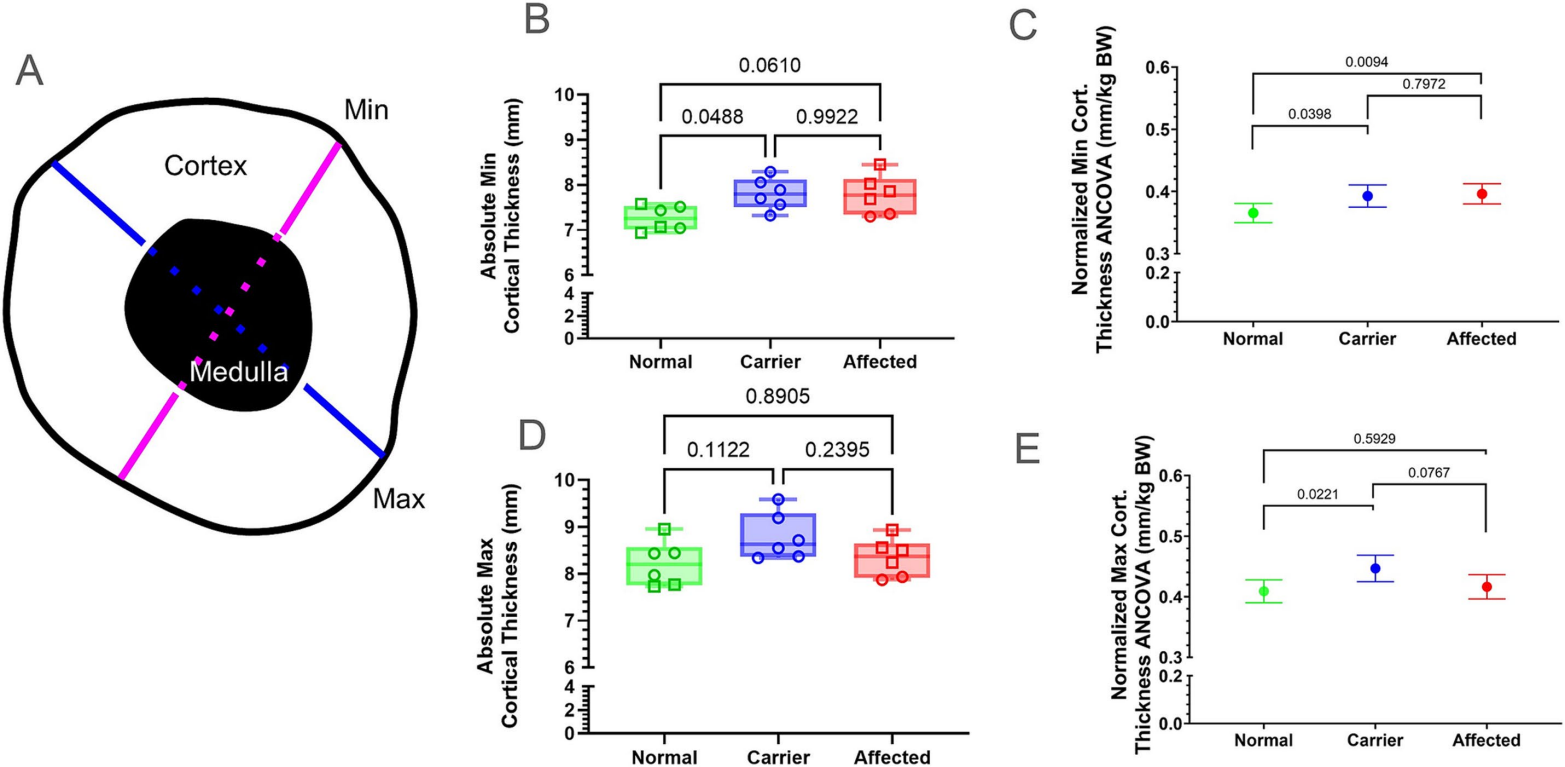
Cortical thickness (CorT) measured at the mid-diaphyseal region. **(A)** Graphical representation of CorT measurement at the mid-diaphyseal minimum (min, pink) and maximum (max, blue) cross-sectional diameter. Min and max CorT were calculated by subtracting the medullary width (dashed line) from the total diameter. **(B,D)** Raw values for min CorT **(B)** and max CorT **(D)** were in millimeters (mm). Results were analyzed via Oneway ANOVA and plotted with individual dog values. KG, kilogram; BW, body weight; Green = Normal, Blue = Carrier, Red = GRMD affected dogs, male (square), female (circle). **(C,E)** Multiple linear regression **(**ANCOVA) plus a normality test (Gaussian) were performed for min **(C)** and max CorT **(E)**, adjusting for covariates’ genotype, weight, age, and sex. The interpolated mean was graphed and included min and max. Min, minimum; BW, body weight; Cort., cortical; mm, millimeters; KG, kilogram.

We measured absolute min and max CorT at the mid-diaphyseal femur (Figure 2A) and compared both absolute values and those normalized by BW (Supplementary Table 1). Normalized min CorT was significantly smaller in normal vs. GRMD and carriers (*p* < 0.05 for both; Figures 2B,C), while absolute min CorT was smaller in normal vs. carriers only. Neither absolute nor normalized min CorT values differed between GRMD vs. carriers (Figures 2B,C). Only normalized values for max CorT were greater in carriers vs. normal (*p* < 0.01 for both; Figures 2D,E).

### 3.4 Cortical density of the femur (CorDen)

We measured CorDen in the same mid-diaphyseal region as CorT. Values were not different between groups (Supplementary Figure 3; Supplementary Table 1).

### 3.5 Muscle volume

**Figure 3:**
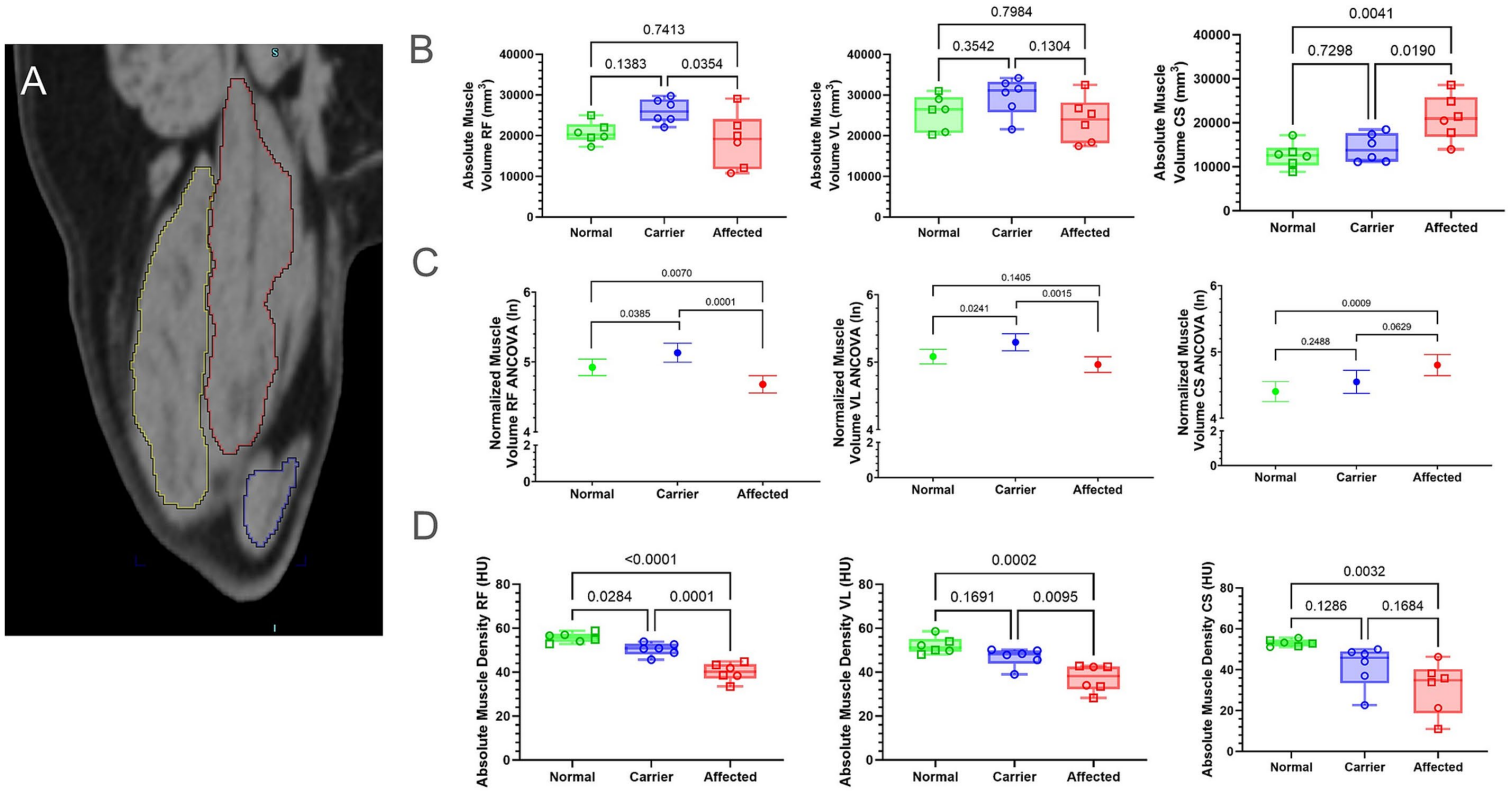
Muscle volume and density differed between groups. Muscle density did not pass normality tests for all muscles; therefore, all volume and density measurements were log-normal (ln) transformed; raw data found in Supplementary Figure 3. **(A)** Muscle volume of the CS (blue outline), RF (red outline), and VL (yellow outline) were calculated by interpolation of whole muscle ROI from manually segmented muscle slices using Inveon internal software. **(B)** Raw values for muscle volume were in millimeters (mm). Results were analyzed via One-Way ANOVA and plotted with individual dog values. **(C)** Multiple linear regression **(**ANCOVA) plus a normality test (Gaussian) were performed, adjusting for covariates’ genotype, weight, age, and sex. The interpolated mean was graphed and included min and max. **(D)** Raw values for muscle density were in HU. Results were analyzed via One-Way ANOVA and plotted with individual dog values. CS, cranial sartorius; RF, rectus femoris; VL, vastus lateralis; ROI, region of interest; HU, Hounsfield units; FL, femur length; KG, kilogram, BW, body weight, Green, Normal, Blue = Carrier, Red = GRMD affected dogs, male (square), female (circle).

We measured and compared both absolute (mm^3^) and normalized volume (volume divided by FL [mm^2^]) for the CS (Figure 3A, blue outline), RF (Figure 3A, red outline), and VL (Figure 3A, yellow outline) muscles (Figure 3; Supplementary Figure 3; Supplementary Table 2). The absolute CS muscle volume was significantly larger in GRMD dogs compared to normal (*p* < 0.01) and carriers (*p* < 0.05), while normalized volume only differed between GRMD and normal dogs (*p* < 0.001; Figures 3B,C). In carriers, both absolute and normalized RF volumes were significantly larger than GRMD (*p* < 0.05; *p* < 0.0001) and normalized RF volume was significantly larger than normal (*p* < 0.01; Figures 3B,C). Only normalized RF muscle volume differed when comparing GRMD and normal dogs (*p* < 0.05; Figures 3B,C). Normalized VL volume was greater in carriers vs. GRMD (*p* < 0.01) and normal (*p* < 0.05), while GRMD vs. normal did not differ (Figures 3B,C).

### 3.6 Muscle density

We measured muscle density/attenuation in HU, an indication of density (24) in the same ROI used for muscle volume (Figure 3D; Supplementary Figure 4). GRMD dogs had significantly lower attenuation than normal and carrier dogs in the RF and VL muscles (Figure 3D). Unexpectedly, carriers had significantly lower HU than normal dogs in the RF, but not the VL or CS. In general, normal dogs had the highest HU in all muscles compared to GRMD and carrier values were intermediate to the normal and GRMD dogs.

### 3.7 Correlations

**Figure 4:**
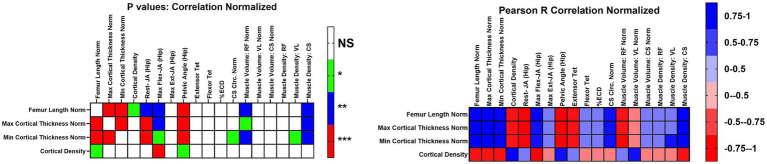
Correlations of functional outcome measures for affected dogs. Correlations for values normalized to body weight or femur length. Normalized correlations were reported as Pearson R coefficients, and *p* values were adjusted for multiple testing. Values were R^2. Max, maximum; Min, Minimum; Rest-JA, resting hip joint angle; Max flex JA, Maximum hip joint angle; Max Ext JA, aximum hip joint angle extension; Extensor Tet, extensor tetanus; Flexor Tet, Flexor tetanus; ECD, Eccentric contraction decrement; Cirm, Circumference; RF, Rectus femoris; VL, Vastus lateralis; CS, Cranial sartorius; Norm, Normalized; NS, no significance.

We analyzed the data for new correlations between CT bone measurements vs. our established muscle function measurements (eccentric contraction decrement [ECD], tibiotarsal tetanic extension [Ext-Tet] and flexion tetanic torque [Flex-Tet], joint angles, and CS circumference) (Supplementary Table 3; Figure 4). We focused on those that were strongly correlated (R^2 > 0.75 or < −0.75), novel, and related to bone in GRMD. For BW normalized data, FL was strongly and positively correlated with CorT. FL was also highly correlated with resting hip joint, max flexed hip joint, and pelvic angle. Finally, FL was also correlated with CS muscle density. Max and min CorT were strongly and positively correlated with CS muscle density, and negatively correlated with resting hip joint angle and pelvic angle. Both max and min CorT were negatively correlated with normalized RF volume, but only min CorT was strongly correlated. Max CorDen was strong and negatively correlated with maximum hip flexion angle.

## 4 Discussion

While bone health has been assessed in DMD and the *mdx* model (8, 9, 12–15), minimal studies have been completed in the canine DMD model (16). In this study, we used CT and showed that GRMD dogs had some changes in bone measurements compared to normal (increased normalized min CorT) and carrier (increased normalized FL) dogs and some of those changes correlated with functional muscle measurements. We acknowledge these results should be interpreted cautiously due to variable age ranges and mixed gender groups. We initially hypothesized that GRMD dogs would have findings consistent with reduced bone health, in keeping with DMD boys (8, 12). However, in GRMD, FL, CorDen, and max CorT were not different and normalized min CorT was slightly greater (thicker) compared to normal animals; normalized FL was slighter larger in GRMD compared to carriers. These results could be interpreted, in part, as preserved bone health in GRMD dogs. This contrasts with the reduced bone length and density in DMD, especially with corticosteroid treatment (8, 9, 12) and is in keeping with the tendency for many GRMD dogs to stabilize after the first 6 months of life (3). We again acknowledge the age range was quite variable in our study; the average age of normal, carrier, and GRMD dogs were, respectively, mismatched at 17, 41.7, and 45 months, with a range of up to 40+ and 70+ months for normal and carrier/GRMD dogs. As such, the normal dogs may not have matured completely from an orthopedic standpoint. Longitudinal studies of bone development in dogs are scarce; one study looking at growth factors in several large breed dogs found that bone measurements stabilized by 1 year (25). That said, we did not observe any incomplete growth plates in the femur of any dogs, making it less likely that FL would change in the groups. *Mdx* mice also had evidence of paradoxically increased bone health in the form of longer femurs and greater femoral bone mineral density (15) compared to controls, in contrast to decreased bone mineral density in DMD boys (8, 26, 27). Even in the face of muscle weakness, GRMD dogs retain ambulation and mobility without fractures (3) potentially owing, in part, to preserved normalized FL and mid-diaphyseal min CorT.

To the authors’ knowledge, bone health has not been studied in carriers of DMD mutations in either humans or animal models. Since carrier dogs maintain a functionally normal skeletal muscle phenotype, we hypothesized that bone and muscle measurements would be similar to those in normal animals. In humans, carriers are *de facto* female, so gender matching, as in the other two groups is not possible except in the ultra-rare case of X-inactivation leading to dystrophic symptoms. In this study, due to the nature of breeding pairs (affected males bred to carrier females), groups contained normal and GRMD females, so direct comparisons were made between all three groups. The stature of female dogs, especially in large breeds, is shorter than males (28), which was observed in our study with carriers FL trending smaller than normal dogs and being significantly smaller than GRMD dogs when normalized to BW. Again, due to age differences, these results should be interpreted with caution. While we have not detected muscle function differences between male and female affected dogs in past studies, the difference in gender distribution between groups (all female carriers vs. 4 M/2F in normal and GRMD groups) may explain at least some difference in bone and muscle density values.

We found a number of correlations between CT measured bone and muscle measurements and established functional measurements in GRMD, but for the discussion, we focused on novel bone related-correlations. FL, max and min CorT were strongly and negatively associated with both resting hip joint angles and pelvic angle, such that animals with more acute resting hip joint and pelvic angles associated with thicker bone. GRMD dogs typically develop larger (more obtuse or restricted) resting hip joint angles and pelvic angles as part of postural changes of disease progression. These findings suggest more severely affected animals may have thinner femurs. Min CorT differed between normal and both GRMD and carrier dogs which could suggest a compensatory increase in overall bone thickness in GRMD and carrier dogs. Both absolute and normalized min CorT were also significantly higher in carrier dogs than normal, but not different between carriers and GRMD in either absolute or normal values. Carriers and GRMD groups were also older than the normal group and it is possible this represents an age-related thickening or altered muscle forces in carriers, as well. Alternatively, in carriers, it could represent a pregnancy-related change; both humans and rats have increased cortical bone volume at the end of pregnancy, with a return of volume in the post lactational period (29). Muscle density of the GRMD CS muscle was also strongly and positively correlated with FL and min/max CorT while min CorT was negatively associated with greater RF muscle volume. Previous studies comparing GRMD to normal animals have found extensor quadriceps (including the VL and RF) progressively wastes away while the CS hypertrophies more in severely affected dogs. Both the RF and CS function as an extensor and somewhat as a hip flexor (a mixed function muscle) (5). These correlations could further support decreased bone thickness in animals with more severe disease.

We found strong negative correlation between CorDen and max flexed hip joint angle, such that animals with greater (more obtuse or restricted) hip flexion angles, as seen in GRMD disease progression, had decreased bone density. We would like to note that we did not find differences in CorDen when comparing the groups. The GRMD dogs in this study had a wide range of measured bone densities, in keeping with the phenotypic variability seen in this model. It is possible that statistical differences in bone density could be detected in a larger cohort. Additionally, the femur may not be the optimal bone in which to measure differences in CorDen due to its thickness and extensive weight bearing properties. In DMD, the spinal column is often used to study osteopenia, though the femur also showed changes (12). Examination of vertebrae in GRMD dogs should also be assessed.

In addition to bone health, we looked at muscle health markers, namely volume and density. While past studies have demonstrated differences between normal and GRMD dogs, to the authors’ knowledge, carrier muscle health has not previously been included. Skeletal muscle fibers are syncytia formed by the fusion of multiple cells, so in carriers, nuclei carrying a functional copy of the DMD gene are expected to compensate for those without, except in cases of skewed X-inactivation. Thus, we hypothesized that carrier skeletal muscle would be similar to normal muscle. The CT findings showed surprising changes in muscle density and volume in the carrier dogs, and these differences varied between muscle groups.

As previously reported, we found significant differences in muscle volumes between normal and GRMD dogs (5, 6, 30). While some studies have shown that muscle volume normalized to FL may be more accurate (17), other studies simply compared direct volume among age matched dogs (30). In this study, we examined both normalized and absolute muscle volumes. The absolute muscle volume in the CS was significantly larger in GRMD dogs than either carriers or normal animals. This finding aligns with previous studies, which suggest that the CS is relatively spared and has true hypertrophy (5, 6). The CS muscle of neonatal GRMD dogs endures extensive necrosis and then regenerates, undergoing intense hypertrophy under the control of several hypertrophy-related pathways (6).

Surprisingly, the RF and VL volume of carriers were increased compared to GRMD and/or normal. Previous studies comparing GRMD to normal animals have found that the RF has increased damage because it functions both as an extensor and somewhat as a hip flexor (a mixed function muscle) (5). While muscle hypertrophy is also seen in certain flexor muscles in GRMD, the extensor quadriceps (including VL muscle) progressively wastes away (5). Due to its dual extensor/flexor function, the apparent RF hypertrophy in the carrier animals may be a physiological response to initial increased damage in the heterozygous fibers. This may lead to a hypertrophic repair response that does not undergo the subsequent degree of atrophy documented in older GRMD animals. Obviously, further studies of carrier RF muscle would be needed to confirm this hypothesis.

Muscle attenuation (density) also differed in GRMD, carrier, and normal animals. Attenuation corresponds to fat content of muscle, with lower attenuation values (measured in HU) corresponding with increased fat (24). As might be expected, GRMD had the lowest values, carriers had an intermediate phenotype, while normal dogs had the highest HU values in all muscles. These data further support the idea of damage and muscle wasting in this flexor/extensor muscle group. Because the carrier group is older than the normal dogs, decreasing muscle attenuation with age or overall body fat is a possibility. One study comparing CT muscle attenuation between old (>8y) and young (1 to 5y) Labradors found lower mean attenuation in the older dogs and a negative correlation with age. Only one of our carriers fell outside the young dog range for that study, and there was wide overlap of attenuation values in the original study, but it suggests inclusion of more and older animals would be useful in determining the significance of these results (31). GRMD and DMD carriers are not often included in studies, as they are considered asymptomatic. However, significant histologic lesions in the heart of GRMD carriers (11) and our findings demonstrate manifestations in some muscles.

### 4.1 Limitations

The age differences have been discussed above and are a major limitation of this study. Additionally, the CT scans and force measurements were not collected simultaneously (on the same day) although they were collected within a month in most cases and were collected as part of previous PET-CT studies in this group of dogs. Therefore, the study was limited by the CT scans available. While DEXA can be a more precise measurement of bone density, it is not widely available. DEXA scanning requires specialized equipment and training, and as a calculated measure requires correction when dealing with disease states like GRMD that directly affect body surface area relative to FL. Computed tomography scans are widely available and commonly used in diagnostics, and comparisons of DEXA with CT scans showed that measurements made with these two techniques correlated (32). To better characterize bone health in GRMD and whether therapies could be assessed, systematic analyses of CT, DEXA, biomechanical properties, histopathologic features, and gene expression on bones taken at necropsy are needed.

In people, DMD is almost exclusively a disease of hemizygous males. In GRMD colonies, breeding affected males to carrier females allows for assessment of homozygous GRMD females, maximizing the number of affected dogs, and most studies utilize mixed gender groups. Previous studies have not shown a difference between these groups in functional measures (22, 33), but it is possible that hormonal differences influence some aspects of disease progression. Similarly, carriers of this X-linked disease are by definition female. Differences between the carrier group, GRMD and normal could be influenced by this fact.

## 5 Conclusion

Some of the bone measurements analyzed here are altered in affected dogs. Correlation with other functional outcome measures suggest that the preservation of bone measures may contribute to a milder phenotype. Results should be interpreted cautiously due to age range variability and mixed gender between groups. Further studies should comprehensively track bone health in gender and age matched normal, carrier, and GRMD dogs in order to further confirm these findings.

## Authors note

A portion of the data was presented at the National Veterinary Scholars 2019 Symposium Poster Session and the Veterinary Medical Summer Research Training Program (VMSRTP) 2019 Conference at Texas A&M University School of Veterinary Medicine & Biomedical Sciences.

## Data Availability

The original contributions presented in the study are included in the article/Supplementary material, further inquiries can be directed to the corresponding authors.

## Supplementary material

SUPPLEMENTARY FIGURE 1 Weight and age comparisons between groups. **(A)** Raw values were in kilograms (kg); min, max, and mean. Results were analyzed via One-Way ANOVA and plotted with individual dog values. **(B)** Individual dog weights by age. KG, kilogram; M, months; Green, Normal; Blue, Carrier; Red, GRMD affected dogs, male (square); female (circle).

SUPPLEMENTARY FIGURE 2 Cortical density (CorDen) comparisons between groups. Raw values were in Hounsfield units (HU). Results were analyzed via One-Way ANOVA and plotted with individual dog values. Green, Normal; Blue, Carrier; Red, GRMD affected dogs, male (square), female (circle).

SUPPLEMENTARY FIGURE 3 Log scale and log transformed muscle volume measures. **(A)** Raw values were placed in a log-scale due to not having Gaussian distribution. Results were analyzed via One-Way ANOVA and plotted with individual dog values. **(B)** Raw values were transformed to ln; min, max, and mean. The volumes did not pass normality. Results were analyzed via one-way ANOVA and plotted with individual dog values. Green, Normal; Blue, Carrier; Red, GRMD affected dogs, male (square), female (circle).

SUPPLEMENTARY FIGURE 4 Log scale and log transformed muscle volume density measures. **(A)** Raw values were placed in a log-scale due to not having Gaussian distribution. Results were analyzed via One-way ANOVA and plotted with individual dog values. **(B)** Raw values were transformed to ln. The volumes did not pass normality. Results were analyzed via one-way ANOVA and plotted with individual dog values. **(C)** Multiple linear regression (ANCOVA) were performed on ln data, adjusting for covariates’ genotype, weight, age, and sex. The interpolated mean was graphed and included min and max. Green, Normal; Blue, Carrier; Red, GRMD affected dogs, male (square), female (circle).
