# Supplementary figures and images for: Bone measurements interact with phenotypic measures in canine Duchenne muscular dystrophy
Source: Front Vet Sci. 2025 Jan 6;11:1339833. doi: 10.3389/fvets.2024.1339833 (PMC11744001; doi:10.3389/fvets.2024.1339833)

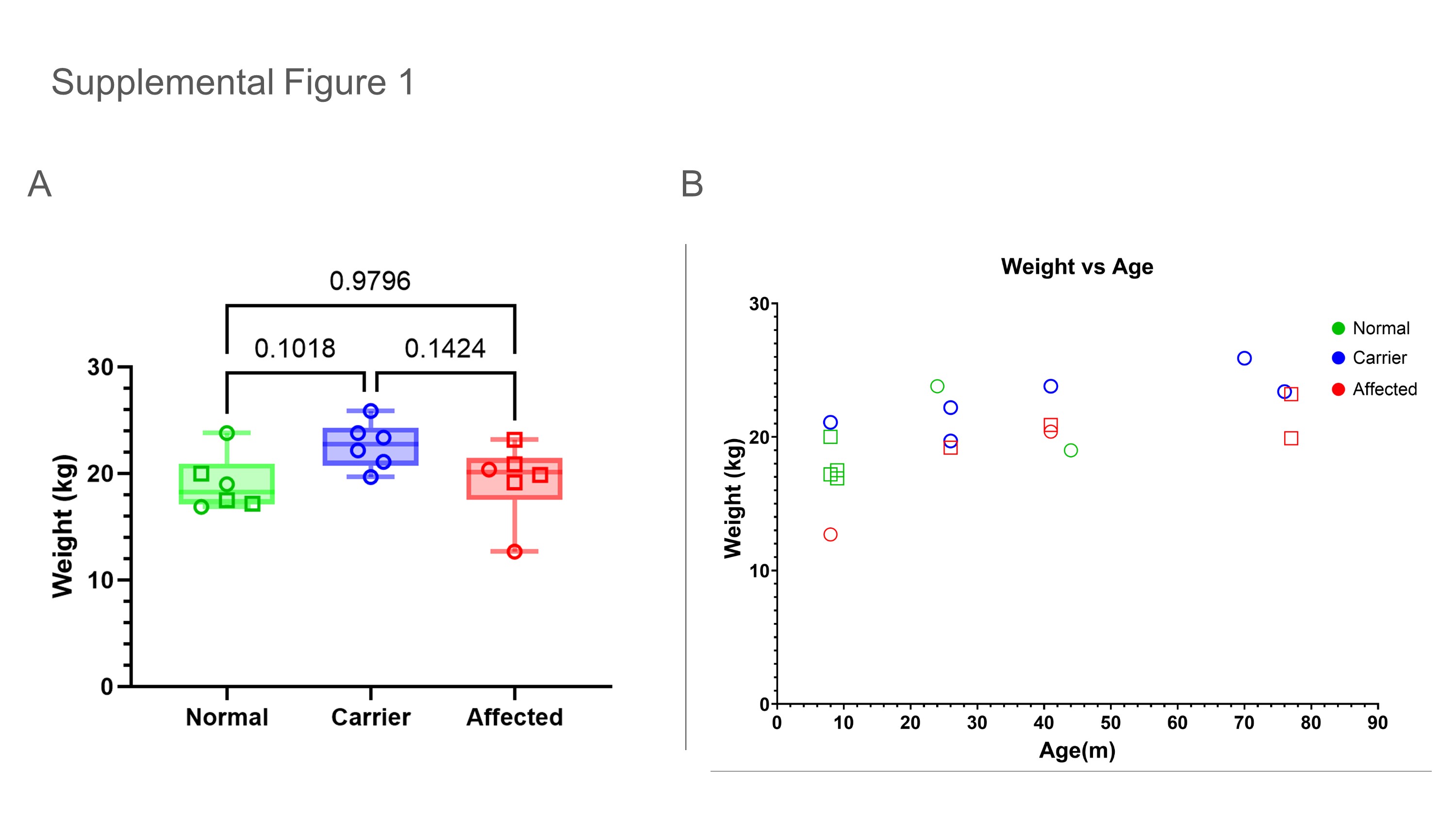

Supplement: SUPPLEMENTARY FIGURE 1 — Weight and age comparisons between groups. (A) Raw values were in kilograms (kg); min, max, and mean. Results were analyzed via One-Way ANOVA and plotted with individual dog values. (B) Individual dog weights by age. KG, kilogram; M, months; Green, Normal; Blue, Carrier; Red, GRMD affected dogs, male (square); female (circle). [file Image_1.jpeg]

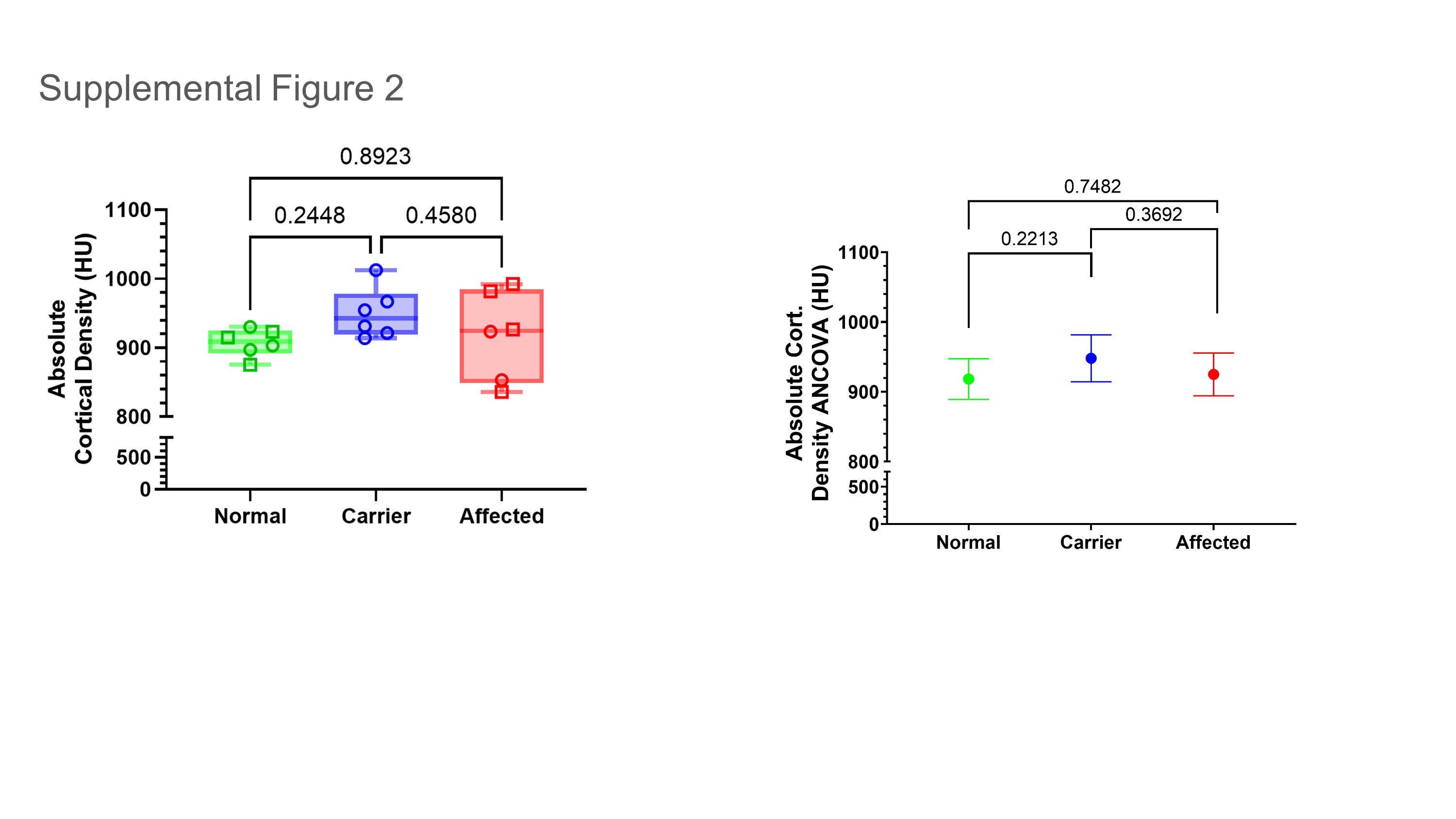

Supplement: SUPPLEMENTARY FIGURE 2 — Cortical density (CorDen) comparisons between groups. Raw values were in Hounsfield units (HU). Results were analyzed via One-Way ANOVA and plotted with individual dog values. Green, Normal; Blue, Carrier; Red, GRMD affected dogs, male (square), female (circle). [file Image_2.jpeg]

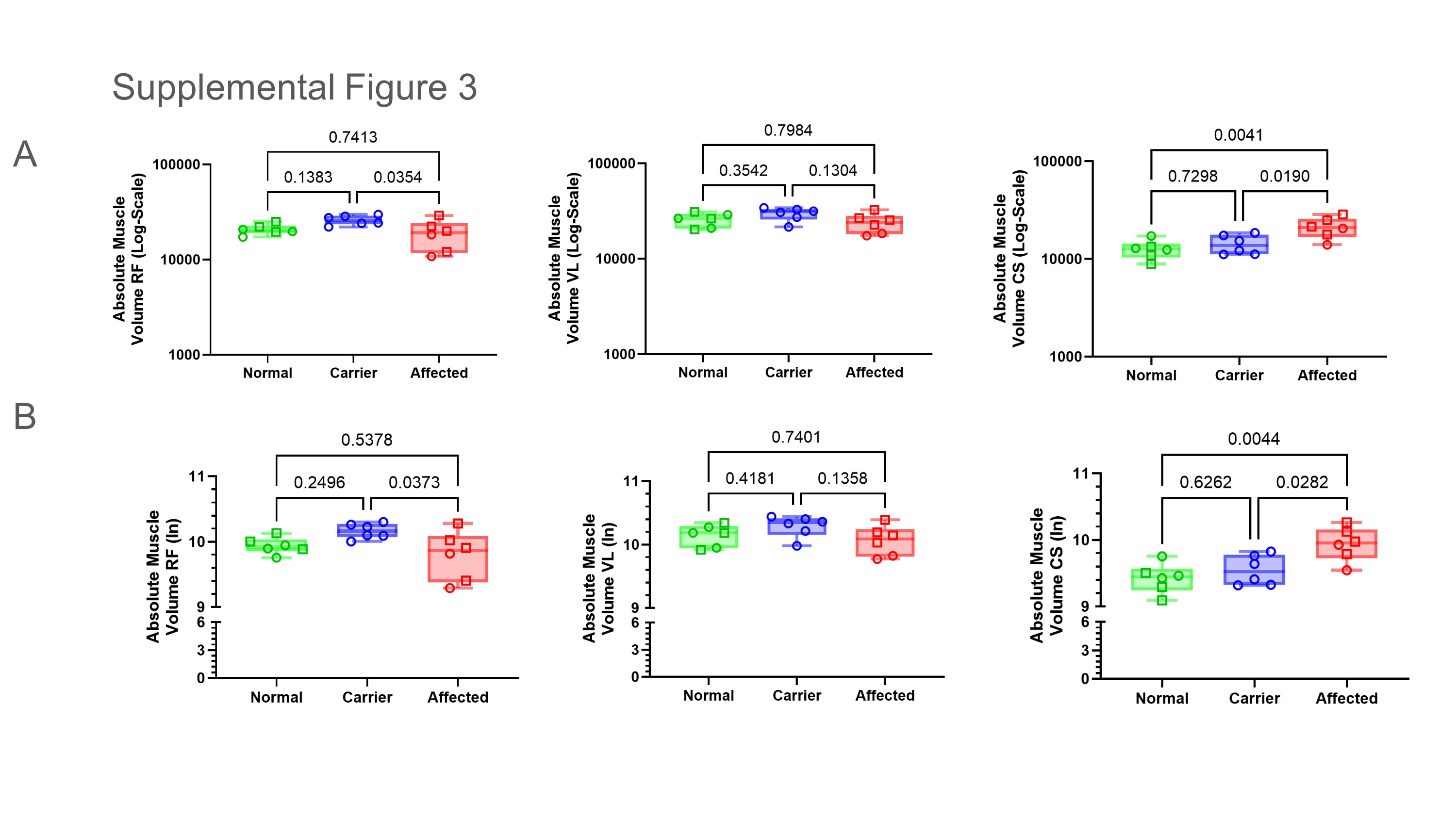

Supplement: SUPPLEMENTARY FIGURE 3 — Log scale and log transformed muscle volume measures. (A) Raw values were placed in a log-scale due to not having Gaussian distribution. Results were analyzed via One-Way ANOVA and plotted with individual dog values. (B) Raw values were transformed to ln; min, max, and mean. The volumes did not pass normality. Results were analyzed via one-way ANOVA and plotted with individual dog values. Green, Normal; Blue, Carrier; Red, GRMD affected dogs, male (square), female (circle). [file Image_3.jpeg]

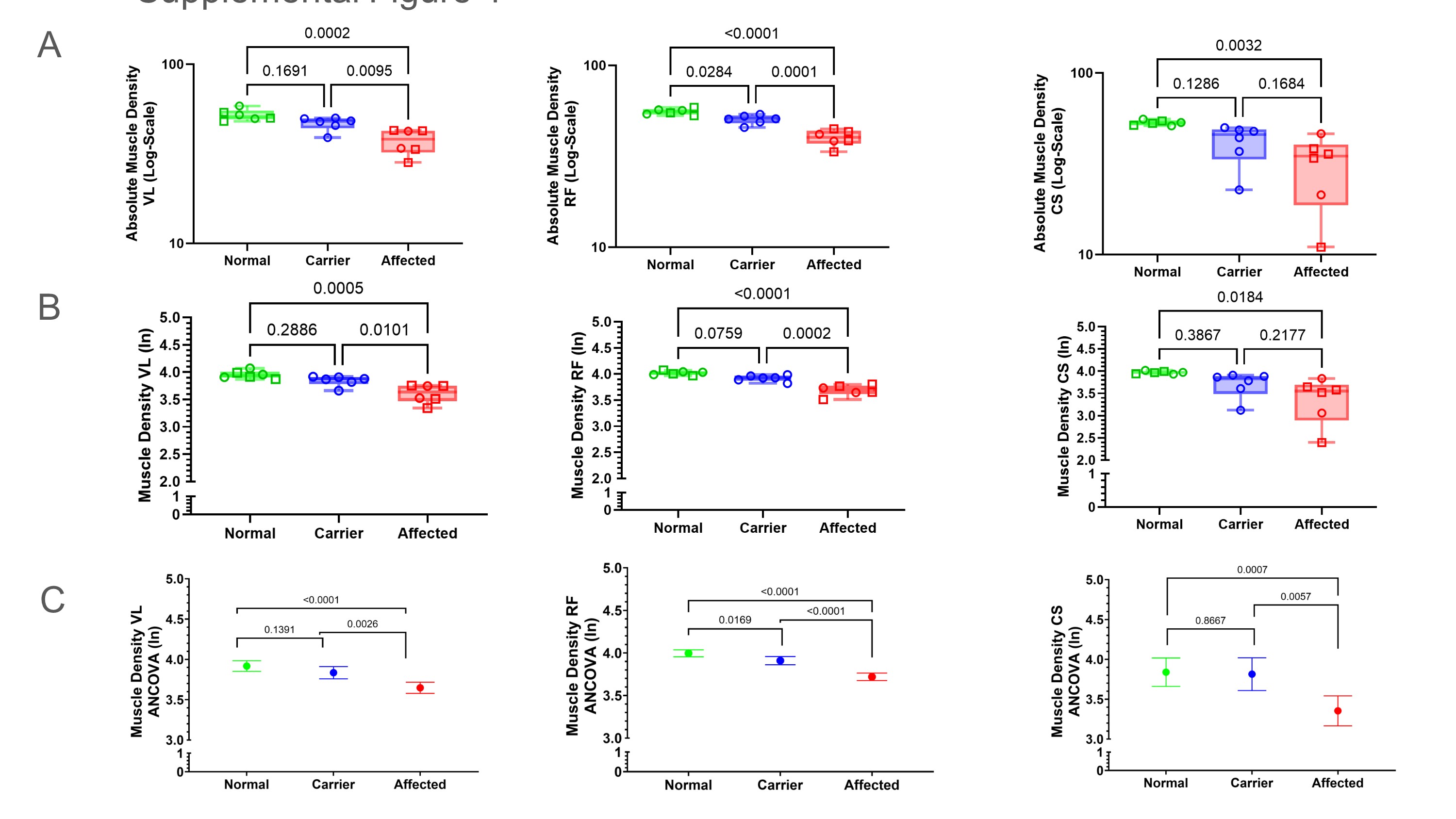

Supplement: SUPPLEMENTARY FIGURE 4 — Log scale and log transformed muscle volume density measures. (A) Raw values were placed in a log-scale due to not having Gaussian distribution. Results were analyzed via One-way ANOVA and plotted with individual dog values. (B) Raw values were transformed to ln. The volumes did not pass normality. Results were analyzed via one-way ANOVA and plotted with individual dog values. (C) Multiple linear regression (ANCOVA) were performed on ln data, adjusting for covariates’ genotype, weight, age, and sex. The interpolated mean was graphed and included min and max. Green, Normal; Blue, Carrier; Red, GRMD affected dogs, male (square), female (circle). [file Image_4.jpeg]
